# Design and Demonstration of Compact and Lightweight Imaging Spectrometer Based on Schwarzschild Reflector Systems Using Commercial Off-the-Shelf Optics

**DOI:** 10.3390/s25247497

**Published:** 2025-12-10

**Authors:** Shuai Yuan, Min Huang, Xuehui Zhao, Fengkun Luo, Han Gao, Zixuan Zhang, Wenhao Zhao, Guangming Wang, Zhanchao Wang, Peng Jiang, Wei Han, Lulu Qian, Guifeng Zhang

**Affiliations:** 1 Aerospace Information Research Institute, Chinese Academy of Sciences, Beijing 100094, China; 2 State Key Laboratory of Remote Sensing and Digital Earth, Beijing 100094, China; 3 Institute of Stealthy Engineering Technology, Beijing 100094, China

**Keywords:** hyperspectral imaging, Schwarzschild reflector systems, commercial off-the-shelf optics, precision agriculture, environmental monitoring

## Abstract

Hyperspectral imaging systems are widely used in precision agriculture, environmental monitoring, and mineral exploration. However, current systems often suffer from high cost, large size and weight, and considerable system complexity, which hinder their widespread deployment. To overcome these limitations and achieve a better balance between performance, cost, and portability, this work aims to develop a compact, cost-effective visible-to-near-infrared (VNIR, 400–1000 nm) hyperspectral camera based on Schwarzschild configuration and commercial off-the-shelf (COTS) components. The development followed a comprehensive methodology encompassing theoretical design, simulation, prototype assembly, and performance testing. The all-reflective optical system effectively eliminates chromatic aberration and minimizes energy loss, achieving an integration time as short as several milliseconds and a push-broom frame rate of 200 fps. The optical design leveraged optical path length theory and the unobscured Schwarzschild structure to optimize off-axis mirrors and a plane grating. Optical performance was optimized and verified using simulations, which confirmed that spot sizes at all field positions were highly concentrated and that critical distortions such as smile and keystone were controlled within several pixels. A prototype was assembled on a precision optical bench using multi-axis adjustable mounts and then integrated into a precisely machined housing, achieving a total weight less than 2 kg. Calibration verified a spectral coverage of 400–1000 nm and a resolution of 5 nm. Imaging experiments demonstrated the system’s ability to resolve subtle spectral features, successfully distinguishing different vegetations and artificial materials based on their spectral signatures—particularly the strong NIR (780–1000 nm) reflectance of vegetation versus synthetic green materials. The camera offers a high-performance, low-cost solution suitable for applications including precision agriculture, environmental monitoring, mineral exploration, and others.

## 1. Introduction

Hyperspectral imaging (HSI) is a powerful remote-sensing technology that captures high-resolution spatial and spectral data across hundreds of contiguous bands, particularly in the VNIR (400–1000 nm) range. This capability enables precise identification of material composition, physiological states, and pathological conditions through unique spectral signatures [1,2,3]. In precision agriculture, HSI has emerged as a non-destructive tool for early detection of crop diseases, nutrient deficiencies, and water stress, with machine learning models achieving over 90% classification accuracy [4,5,6]. Its applications extend to mineralogical mapping [7,8,9] and biomedical diagnostics [10,11,12], where it supports tumor detection and surgical guidance with high specificity. Despite its versatility, HSI faces challenges including high sensor costs, data dimensionality issues, and sensitivity to environmental variations, driving the need for portable and cost-effective solutions [13,14].

The growing demand for compact, lightweight HSI devices operating in the VNIR band is fueled by applications in unmanned aerial vehicle (UAV)-based remote sensing, real-time field diagnostics, and industrial monitoring [15,16]. Traditional HSI configurations such as Czerny-Turner or prism spectrometers often contend with optical aberrations including smile and keystone distortions, limited field of view, and chromatic errors [17,18,19]. In contrast, the Schwarzschild optical design offers significant advantages including minimal obscuration, excellent aberration correction, and high optical efficiency. It has been extensively studied and refined to meet the growing demand for compact, high-performance imaging systems in various applications [20,21]. By employing a symmetric two-mirror system combined with a plane grating, Schwarzschild spectrometers achieve compact form factors with reduced astigmatism [22,23,24]. For example, Qingsheng Xue proposed a modified Schwarzschild imaging spectrometer in 2013, using three nonconcentric aspheric mirrors that achieved low F-number (F/2.5), long slit (13 mm), and broad spectral range (400–1000 nm) [25]. Chen et al. designed a Schwarzschild hyperspectral camera in 2014, showing that astigmatism could be fully eliminated through precise geometric configurations of mirrors and gratings [26]. Liu et al. investigated freeform Schwarzschild imaging spectrometers in 2021, high-lighting that freeform optical surfaces effectively correct off-axis aberrations, improve optical speed, and broaden the system’s FoV. Their design achieved an optical speed of F/2 and a 32 mm slit length [27]. However, current Schwarzschild-based HSI systems are predominantly limited to design-stage concepts or require complex aspheric/freeform surfaces, whose high fabrication costs and extended development cycles have hindered their practical adoption.

In recent years, Commercial Off-The-Shelf (COTS) optical components, which are readily available without custom development, have been widely used in developing hyperspectral imaging systems [28,29,30]. This approach significantly reduces both development time and cost, while maintaining competitive optical performance. Fred Sigernes et al. successfully developed a prototype of push-broom hyperspectral imager with a mass under 200 g using COTS components. These instruments cover the VNIR spectral range, achieving a spectral resolution of 1.4–5 nm. Stability tests demonstrated that the systems are capable of performing push-broom spectral imaging across multiple platforms [28]. In their subsequent work, they transitioned the do-it-yourself (DIY) hyperspectral camera from an S-mount to a C-mount interface, thereby reducing the optical system’s F-number and enhancing its signal-to-noise ratio. The feasibility of deploying the modified system for ocean color monitoring on microsatellites was also demonstrated [29,30,31]. Kimmo Aukusti Riihiaho et al. adopted an existing DIY push-broom HSI design and developed a software solution capable of correcting spectral smile aberration without the need for an optical laboratory. This approach effectively resolves the calibration challenge using tools that are simpler and more cost-effective than conventional methods [32]. Sukrit Thongrom et al. presented a comprehensive framework for developing a compact, cost-effective, and robust hyperspectral camera utilizing COTS components and a volume-phase holographic (VPH) grating. The system demonstrates an ability to effectively discriminate vegetation, water bodies, and architectural structures, resolve atmospheric absorption features, and distinguish various materials under low-light conditions [33].

Despite the existence of several hyperspectral camera designs utilizing COTS components, which are often praised for their cost efficiency and modularity, most of these systems suffer from substantial optical aberrations, extended integration times, and unsatisfactory accuracy in spectral and spatial performance. These shortcomings consequently constrain their practical deployment in demanding applications such as precision agriculture, environmental monitoring, and remote sensing, where broad spectral bands, high fidelity and operational efficiency are critical.

To address the above-mentioned limitations of current COTS-based HSI systems, this work aims to design a hyperspectral camera to achieve a better balance between performance, cost, and portability. The proposed prototype achieves 400–1000 nm spectral coverage with 5 nm resolution, and a total instrument mass of approximately 1.5 kg, making it lightweight enough for both laboratories use and mounting on small UAVs for remote sensing applications. The principal novelty of this work lies in its cost-effective implementation of a Schwarzschild-type hyperspectral camera using COTS spherical mirrors, bypassing the need for the complex and expensive custom aspheric or freeform optics typically required in such designs. By selecting commercial standard mirrors and mount hardware, assembly can proceed in-house at significantly reduced cost compared to bespoke optics systems. The use of a plane reflective grating further simplifies the optical path while improving energy utilization compared to a prism-grism or transmissive approach. Moreover, the broader body of research on hyperspectral systems confirms that instruments weighing ~1 kg and operating in the 400–1000 nm range with ~5 nm FWHM resolution are well-suited to UAV remote sensing of vegetation attributes and chlorophyll indices [34]. Hence, the proposed Schwarzschild camera design aligns with industry-level performance benchmarks while incorporating advanced all-reflective optics to maximize throughput and minimize chromatic limitations, which is suitable for both laboratory settings and aboard UAV platforms for environmental sensing applications.

The organization of this paper is as follows. Section 1 provides an introduction of related topics about COTS hyperspectral camera development. Section 2 focuses on developing and analyzing the proposed COTS-based Schwarzschild hyperspectral camera. Section 3 provides experimental verification of the developed equipment. Section 4 concludes the article with future perspectives.

## 2. Design of Schwarzschild Imaging Spectrometer

The design of a hyperspectral camera primarily involves key steps such as optical design and optimization, optical performance evaluation, and data acquisition software development. The optical design and performance evaluation of this work were primarily conducted in MATLAB (R2023a) and ZEMAX OpticStudio (2023 R1.00, 64-bit), while the data acquisition and analysis software was developed using Python 3.8 programming language. Given the project’s emphasis on utilizing COTS optical components wherever possible, the optical design and component selection were mutually constrained. Through an iterative process, continuous trade-offs between cost and performance were made during the design phase, ultimately achieving practically useful performance at relatively low cost.

**Figure 1:**
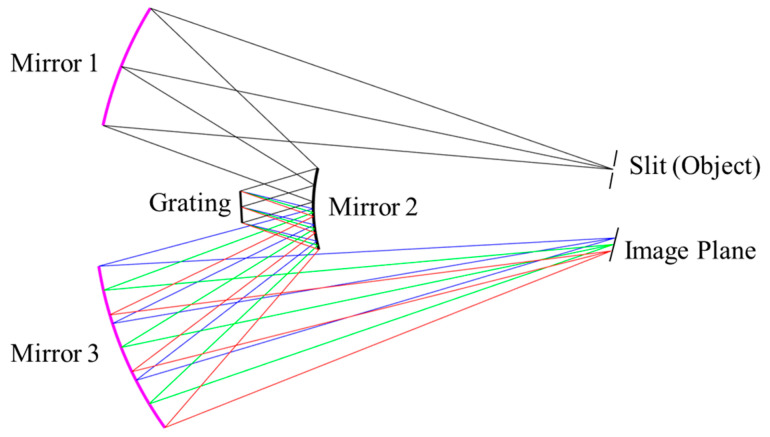
Illustration of Schwarzschild imaging spectrometer consists of three mirrors and a plane blazed grating. The lines in blue, green and red indicates the direction of light with different wavelength.

Generally, a hyperspectral camera can be divided into a collimating system and a focusing system, separated by a grating. Both collimation and focusing can be implemented using either lenses or mirrors. An all-reflective optical system is preferred as it significantly improves energy efficiency. By employing a carefully designed optical scheme with an all-spherical mirror configuration, system costs can be substantially reduced. To achieve the collimating and focusing functions, at least two mirrors are required for each subsystem. Two spherical mirrors with coincident curvature centers make up the most basic two-mirror optical system, which is commonly referred to as the Schwarzschild objective, which was first proposed by German physicist Karl Schwarzschild [35]. By symmetrically arranging two Schwarzschild structures sharing a common convex mirror with identical center and radius of curvature, a Schwarzschild imaging spectrometer consisting of only three reflective mirrors can be realized. Another advantage of this configuration is that it enables the use of a plane grating as the dispersive element, thereby further reducing cost. As shown in Figure 1, incident light entering through the slit is collimated by the upper section of concave mirror 1 and convex mirror 2 and then directed onto the plane grating. The dispersed reflected light is subsequently focused by the lower portion of convex mirror 2 and concave mirror 3, ultimately forming dispersed spectral stripes on the image plane.

The primary aberration in a concentric off-axis two-mirror system is astigmatism. To obtain the initial configuration, the collimating and focusing systems were separated at the grating, and a reverse design methodology was adopted: parallel light was converged to the object point via convex mirror 2 and concave mirror 1. By analyzing the astigmatism-cancelation conditions, the structural parameters of the collimating optical system were derived. Subsequently, under constraints imposed by the grating parameters, the system was made symmetric, and the convex mirrors were merged into a single element, while ensuring non-interference of the optical paths, thereby yielding the final initial optical system. It is important to note that although simultaneously correcting both astigmatism and coma could further improve image quality, it would require two convex mirrors with slightly different dimensions, thereby increasing cost. Therefore, during this design phase, only astigmatism was corrected without addressing coma—a trade-off that would be discussed in detail subsequently.

### 2.1. Analysis of Stigmatic Condition

**Figure 2:**
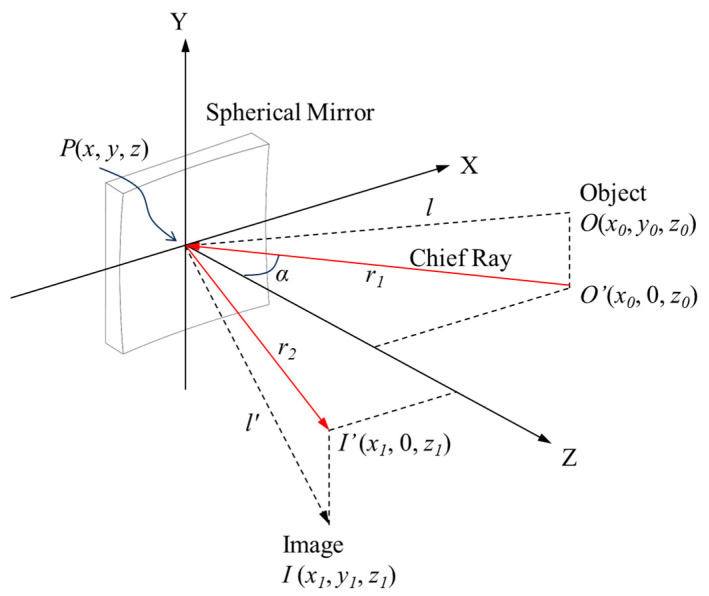
Illustration of the OPL of a spherical mirror with a radius of curvature of R. The arrows indicate the direction of light propagation.

The designing of the Schwarzschild imaging spectrometer was started by analyzing the astigmatism-cancelation conditions for a two-mirror reflective system. According to the optical path length (OPL) theory, the OPL between the object and the image can be used to determine wavefront and ray aberrations in accordance with the image quality standards [26]. As shown in Figure 2, if a light ray coming from the object point *O*(*x*_0_, *y*_0_, *z*_0_) illuminated the point *P*(*x*, *y*, *z*) on the mirror, and was then reflected to the image point *I*(*x*_1_, *y*_1_, *z*_1_), the corresponding OPL is

(1) $$OPL=OP+IP=r_{1}+r_{2}+A_{1}x^{2}+A_{1}^{\prime }y^{2}+A_{2}x^{3}+A_{2}^{\prime }xy^{2}+A_{3}{\left(x^{2}+y^{2}\right)}^{2}$$

(2) $$A_{1}=\frac{1}{2}\left(\frac{\cos^{2}\alpha }{r_{1}}+\frac{\cos^{2}\alpha }{r_{2}}-\frac{2\cos\alpha }{R}\right)$$

(3) $$A_{1}^{\prime }=\frac{1}{2}\left(\frac{1}{r_{1}}+\frac{1}{r_{2}}-\frac{2\cos\alpha }{R}\right)$$

(4) $$A_{2}=\frac{1}{2}\left[\frac{\sin\alpha }{r_{1}}\left(\frac{\cos^{2}\alpha }{r_{1}}-\frac{\cos\alpha }{R}\right)-\frac{\sin\alpha }{r_{2}}\left(\frac{\cos^{2}\alpha }{r_{2}}-\frac{\cos\alpha }{R}\right)\right]$$

(5) $$A_{2}^{\prime }=\frac{1}{2}\left[\frac{\sin\alpha }{r_{1}}\left(\frac{1}{r_{1}}-\frac{\cos\alpha }{R}\right)-\frac{\sin\alpha }{r_{2}}\left(\frac{1}{r_{2}}-\frac{\cos\alpha }{R}\right)\right]$$

(6) $$A_{3}=\frac{1}{8}\left[\frac{1}{R^{2}}\left(\frac{1}{r_{1}}+\frac{1}{r_{2}}-\frac{2\cos\alpha }{R}\right)-\frac{1}{r_{1}}{\left(\frac{1}{r_{1}}-\frac{\cos\alpha }{R}\right)}^{2}-\frac{1}{r_{2}}{\left(\frac{1}{r_{2}}-\frac{\cos\alpha }{R}\right)}^{2}\right]$$

The higher order terms in Equation (1) can be separated into astigmatism, coma, and spherical aberration. The sum *A*_1_*x*^2^ + *A*_1_′*y*^2^ represents astigmatism, with *A*_1_ and *A*_1_′ denote the tangential and sagittal astigmatic image locations, respectively. *A*_2_*x*^3^ + *A*_2_′*xy*^2^ represents coma, and *A*_3_(*x*^2^ + *y*^2^)^2^ is a spherical aberration. According to Equation (1), astigmatism term has the lowest order, so it is responsible for most part of the image degradation. The following work will concentrate on eliminating the astigmatism of the two-mirror system.

**Figure 3:**
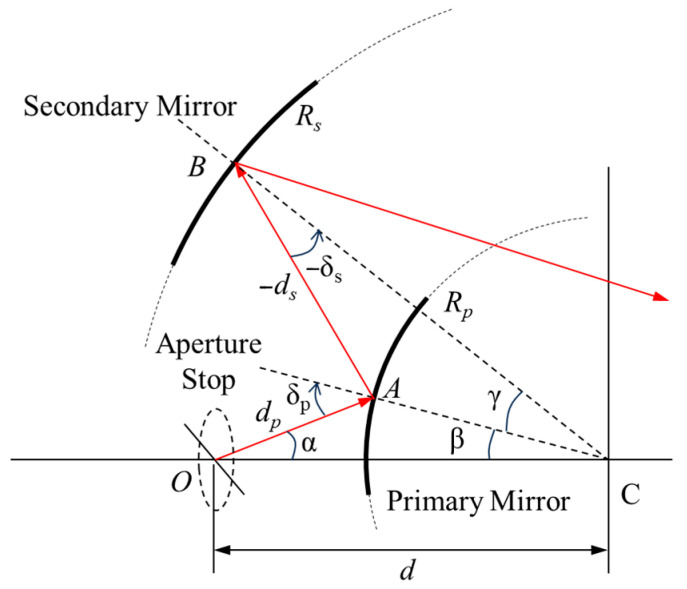
Illustration of the optical path length of a spherical mirror with a radius of curvature of R. The arrows indicate the direction of light propagation.

As previously discussed, an inverse approach was used to analysis the astigmatism-free condition of two-mirror system. In this case, parallel light coming from the aperture stop first arrived at the primary mirror with a radius of curvature of *R_p_*, then reflected to the secondary mirror (*R_s_*), where the light was focused, as shown in Figure 3. According to the OPL theory, the distance (Δ*r*) between the tangential and sagittal image locations of the two-mirror system can be expressed as [26]:

(7) $$\Delta r=R_{p}\sin\delta_{p}[-\frac{\tan\gamma }{2}-\frac{1}{2\left(\frac{1}{\tan\delta_{p}}+\frac{1}{\tan\alpha }\right)}]$$

Let Δ*r* = 0, one can obtain the astigmatism-free condition of two-mirror system:

(8) $$\frac{1}{\tan\delta_{p}}+\frac{1}{\tan\delta_{s}}+\frac{1}{\tan\gamma }=0$$

which can also be written as

(9) $$\tan2\gamma =2(\tan\delta_{p}+\tan\delta_{s})$$

**Figure 4:**
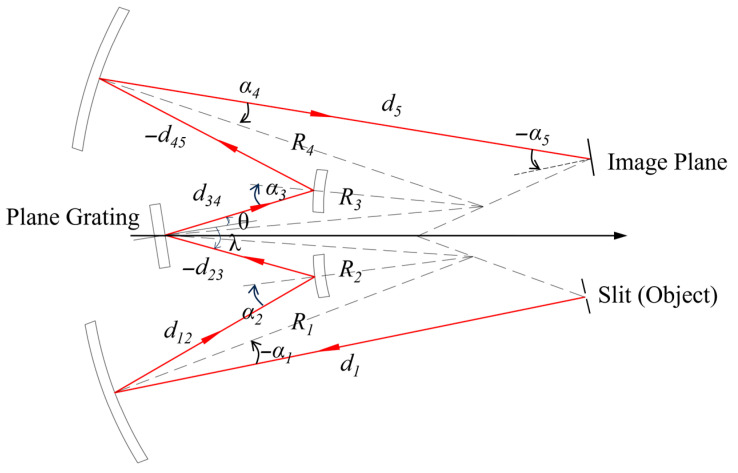
Illustration of the parameters of a Schwarzschild imaging spectrometer based on off-axis dual mirror system. The arrows indicate the direction of light propagation.

Combining two such dual mirror systems with axisymmetric configuration, one could obtain the initial structure of the proposed Schwarzschild imaging spectrometer, as shown in Figure 4.

The coma can be removed only if astigmatism is eliminated. And the coma-free condition is [26]:

(10) $${\left(\frac{R_{2}}{R_{3}}\right)}^{2}={\left(\frac{\cos\lambda }{\cos\theta }\right)}^{3}$$

where *R*_2_ and *R*_3_ are the radius of curvature of the convex mirrors, *λ* and *θ* are the incident angle and diffraction angle of the plane grating, respectively. According to Equation (10), if the coma-free condition is met, there should be a slight difference between the radius of curvature of the convex mirrors. As the two convex mirrors were combined into one piece to reduce the number of components, the coma-free condition could never be met. Finally, a three-mirror Schwarzschild imaging spectrometer with stigmatic condition was achieved.

### 2.2. Initial Structure Design and Component Selection

The key parameters of the proposed Schwarzschild imaging spectrometer are listed in Table 1. In comparison to prior research endeavors, this work has achieved significant advancements, particularly in the domain of spectral coverage. Notably, whereas several commercial hyperspectral imaging systems are constrained to a limited range of 400–900 nm or narrower, the developed instrument successfully extends the operational bandwidth to 400–1000 nm [33]. This enhancement not only improves the capability to capture critical spectral features in the near-infrared region but also significantly broadens the applicability of the system across diverse fields such as precision agriculture, environmental monitoring, and material identification.

The selection of key components began with the CMOS detector. Sony IMX174 (Sony, Tokyo, Japan) was selected considering the required image plane size and wavelength range, which was commonly used in VNIR cameras. A plane reflective blazed grating was chosen as the dispersive element, with a blaze wavelength of 500 nm, dimensions of 15 mm × 25 mm, and grooves oriented along the longer dimension.

The selection of mirror curvature radii depended on the image plane size and the astigmatism-cancelation condition (Equation (9)), while ensuring non-interference in the optical path and minimizing the F-number. A MATLAB (R2023a) program was composed to automate the selection of mirror curvature radii. Based on extensive calculations, an empirical formula was derived: the curvature radius of the concave mirror should be approximately 2 to 2.5 times that of the convex mirror (both taken as absolute values). Finally, concave mirror M1 was selected with a curvature radius of 150 mm and a diameter of 50 mm, while M2 has a curvature radius of 150 mm and a diameter of 100 mm. According to the empirical formula, the convex mirror should have a curvature radius between 60 mm and 75 mm. Due to the limited availability of COTS convex mirrors, a plano-convex lens coated with a reflective layer was used instead. It was noted that a plano-convex lens made of K9 optical glass with a focal length of 125 mm has a convex surface curvature radius of 64.55 mm. Therefore, a plano-convex lens with a diameter of 30 mm and a focal length of 125 mm was selected, with its convex surface coated with a silver film to serve as the convex mirror. A summary of the optical components is provided in Table 2. All the COTS components were provided by Jintao Electro-optics, Co., Ltd., Changchun, China.

After selecting the optical components, another MATLAB program was developed to compute and optimize the initial configuration, mainly according to Equations (9) and (10). Referring to the parameters illustrated in Figure 4, since *R*_2_ and *R*_3_ are combined into a single mirror, parameters such as the mirror curvature radii, *d*_1_, *d*_12_, *d*_45_, *d*_5_, and *α*_1_–*α*_4_ are determined based on the astigmatism-cancelation condition (Equation (9)). However, *d*_23_ and *d*_34_ remain undetermined at this stage, as both the incident and diffracted beams at the plane grating were assumed to be collimated, making these distances theoretically independent of propagation. Nevertheless, *d*_23_ and *d*_34_ do influence the final F-number and image quality: a shorter *d*_23_ helps improve image performance but brings the grating closer to the convex mirror, which requires a larger F-number to avoid optical path obstruction. Therefore, an additional MATLAB program was written to optimize *d*_23_ (with *d*_34_ derived accordingly). Ultimately, all geometric parameters of the optical system were computed and optimized, as summarized in Table 3.

### 2.3. Optical Simulations

**Figure 5:**
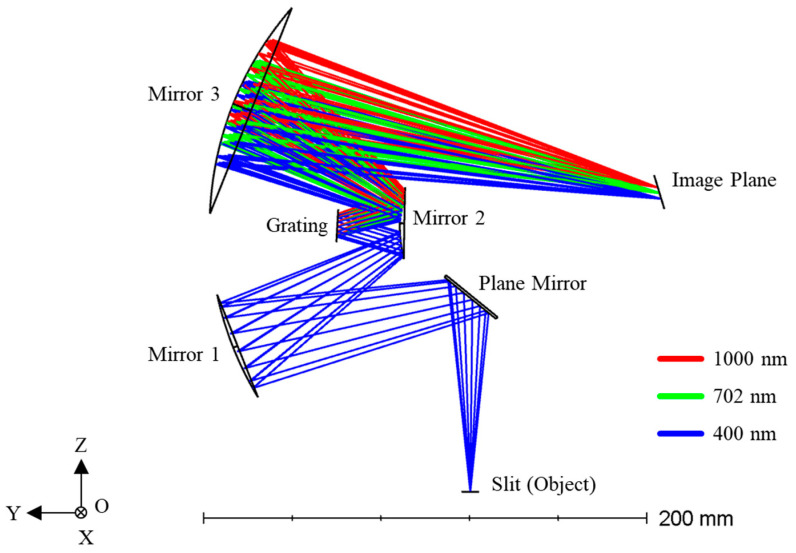
Illustration of the Schwarzschild imaging spectrometer based on off-axis dual mirror system.

Based on the data summarized in Table 3, a hyperspectral imaging camera operating in the 400–1000 nm wavelength range was designed. Figure 5 shows the ray tracing simulation of the system in Zemax OpticStudio (2023 R1.00, 64-bit). The optical path proceeds as follows: incident light first enters the system through the slit (Object), is redirected by a flat folding mirror, and then arrives at the off-axis concave mirror (Mirror 1). Mirror 1 and Mirror 2 collimates the beam and directs it toward the plane grating, which served as the spectral dispersive element. The dispersed beams of different wavelengths are then converged by the concave mirror (Mirror 2) and ultimately form distinct spectral lines on the image plane. The diagram clearly illustrates the ray paths for three characteristic wavelengths: 400 nm (blue), 702 nm (green), and 1000 nm (red).

**Figure 6:**
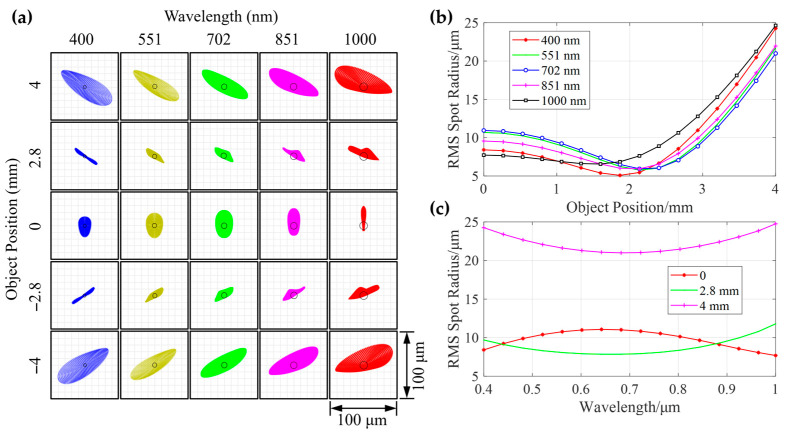
Comprehensive evaluation of optical system performance through spot diagram analysis and RMS spot radius quantification. (**a**) Two-dimensional grid of spot diagrams displaying the distribution and morphology of light spots across wavelengths (400–1000 nm) and object positions (−4 to +4 mm). Note that 702 nm was selected as chief ray since its spot were located at the center. The circles in the middle indicate the Airy disk. (**b**) RMS spot radius as a function of object position (0–4 mm) for five distinct wavelengths. The curves demonstrate the system’s field dependence, with minimal radius variation (<25 μm) indicating effective correction of field curvature and off-axis aberrations. (**c**) RMS spot radius versus wavelength (400–1000 nm) at three object positions (0, 2.8, and 4 mm). The plots reveal spectral uniformity, as all curves maintain low RMS values (<25 μm) across the band.

The optical performance of the system was evaluated through the analysis of spot diagrams and the quantification of the root mean square (RMS) radius across the entire field of view and spectral band. Figure 6a displays spot diagrams of the system at five characteristic wavelengths: 400 nm, 551 nm, 702 nm, 851 nm, and 1000 nm, across the full field of view ranging from −4 mm to +4 mm. The results reveals that the spots at all field positions and wavelengths exhibit excellent concentration and symmetry, with their sizes approaching several pixels. This indicates that aberrations, particularly the stigmatism, have been effectively corrected. The wavelength and field dependence of RMS spot radius is plotted as Figure 6b,c. The curves for different wavelengths demonstrate that the RMS radius remains consistently below 25 µm across the entire field of view. The smoothness and consistent trends of the curves confirm the system’s good field curvature correction capability and uniform imaging performance over the full field. Meanwhile, under various field conditions, the RMS radius is maintained at a low level (<25 µm) throughout the operating band from 400 nm to 1000 nm, with particularly superior performance in the central field and longer wavelength regions. Overall, the data collectively demonstrate that the optical system achieves high imaging quality across the entire target waveband and full field of view. Aberrations are well balanced, fully meeting the stringent requirements of hyperspectral imaging for resolution and spectral fidelity.

**Figure 7:**
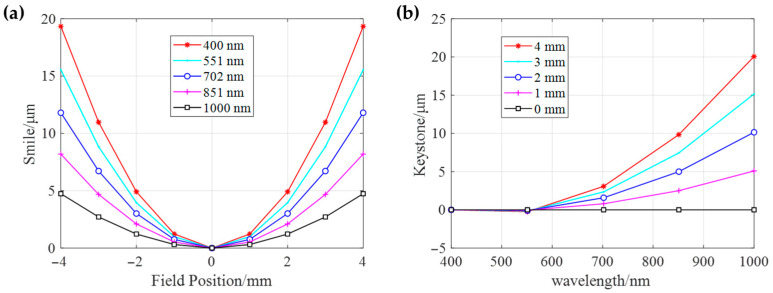
Quantitative analysis of smile and keystone distortions of the hyperspectral imaging system. (**a**) Smile distortion (µm) against field position (mm) for five wavelengths (400–1000 nm), showing symmetric variation with maximal deviation of ~20 µm at field edges. (**b**) Keystone distortion (µm) as a function of wavelength (400–1000 nm) under varying slit thicknesses (0–4 mm), revealing wavelength-dependent spatial shifts below 20 µm. Notice that the keystone of −4–0 mm are inversely symmetry with 0–4 mm, so the plot shows half of the entire field.

Smile and keystone analyses are critically important in the design and performance evaluation of HSI systems, as they directly quantify two key forms of spectral and spatial distortion that degrade data quality. Figure 7a,b presents the quantitative simulation analysis results of the Smile and Keystone of the designed hyperspectral imaging system, respectively. The results indicate that the Smile value is zero at the center of the field of view (0 mm) and increases symmetrically toward the edges (±4 mm), with a maximum absolute value of approximately 20 μm, which is less than the length of four pixels. It is noteworthy that the bending is significantly more pronounced in shorter wavelength bands (e.g., 400 nm) compared to longer wavelength bands (e.g., 1000 nm), indicating a stronger field dependence of chromatic aberration in the short-wave region. Nevertheless, the overall magnitude demonstrates effective control of this aberration. The Keystone value is nearly zero at the short-wave starting end (around 400 nm) and increases monotonically with wavelength, reaching a maximum value of about 20 μm at 1000 nm. Additionally, this distortion exhibits clear field dependence, with larger Keystone values observed at greater field positions (e.g., 4 mm). Results confirms that both Smile and Keystone distortions are effectively constrained within the size of 4 pixels (lower than 20 μm) across the entire waveband and full field of view. This implies that spectral curvature and image point displacement have minimal impact on the spectral purity and spatial registration accuracy of the final hyperspectral data cube.

### 2.4. Tolerance Analysis

Upon completion of the system design, a tolerance analysis must be conducted to determine the required precision of optical and mechanical manufacturing and alignment, and to evaluate the manufacturability and feasibility of the spectrometer system. The analysis was performed using the RMS spot radius as the merit function to assess system performance after tolerance allocation.

Initial tolerance limits were allocated to various optical components in the system, with the image distance selected as a compensator. Tolerance analysis on the RMS spot radius was carried out in optical design software to evaluate multiple tolerance items of the spectrometer system, including, but not limited to, radius of curvature tolerance, thickness tolerance, element decenter tolerance, and element tilt tolerance. Tolerance allocation was subsequently performed for the system using the parameters shown in Table 4.

A Monte Carlo analysis with 500 rounds was performed to identify the most sensitive tolerance items that significantly impact system performance. Based on the simulation results and worst-case deviation analysis, it was determined that the decenter and tilt errors of the first and the last mirrors in the system are the most sensitive and have the greatest influence on system performance. In contrast, the decenter errors of the second and fourth mirrors exhibit relatively looser impact on the system. Tolerance reallocation was subsequently carried out.

**Figure 8:**
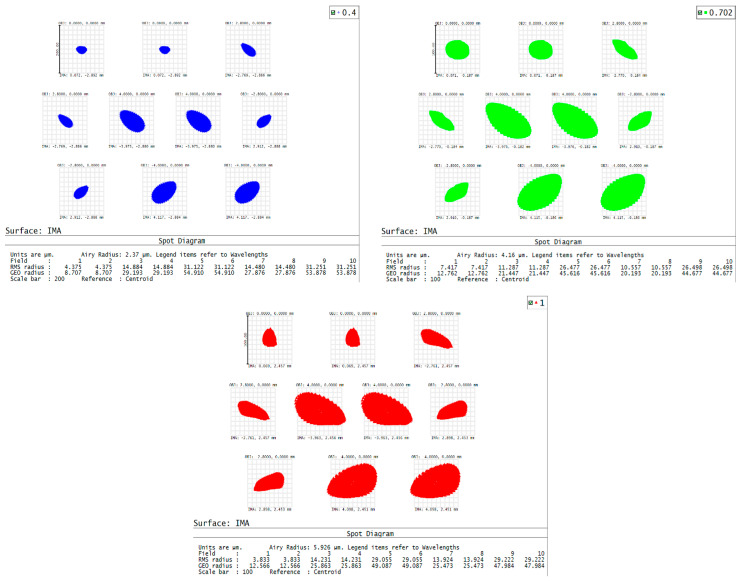
RMS tolerance radius stack-up analysis for the hyperspectral imaging system, showing the spot distributions from a Monte Carlo tolerance analysis, simulating cumulative manufacturing and alignment errors. Elliptical spots in blue, green, and red represent performance at field positions (X, Y) = (4.375, 4.375) mm for wavelengths 400 nm, 702 nm, and 1000 nm, respectively.

The resulting RMS spot radius stack-up analysis after tolerance optimization is shown in Figure 8. Through analysis and adjustment of the tolerance outcomes, the most critical tolerance items of the system were identified. The reallocated tolerance values are summarized in Table 5.

Under the specified system tolerance allocation conditions, the variation in the RMS spot radius remains within an acceptable range. The changes in RMS spot radius and their corresponding probabilities at typical wavelengths are shown in Table 6. The results demonstrate that the specified machining and assembly tolerances are reasonable and feasible.

### 2.5. Data Acquisition Software Design

A data acquisition software for the hyperspectral camera was developed in a Python 3.8 environment, enabling control of camera parameters, data retrieval, and storage for subsequent post-processing. Unlike conventional photography or video applications, the core function of the software involves reading frame-by-frame data and saving it in an uncompressed format. Instead of using video or individual frame saving modes, the software aggregates multiple frames into a single multi-page TIFF file. Upon completion of data acquisition, the raw data undergoes conversion and merging to generate a standard hyperspectral data cube format along with its corresponding header file, ensuring compatibility with analysis tools such as MATLAB R2023a or ENVI 5.6.

**Figure 9:**
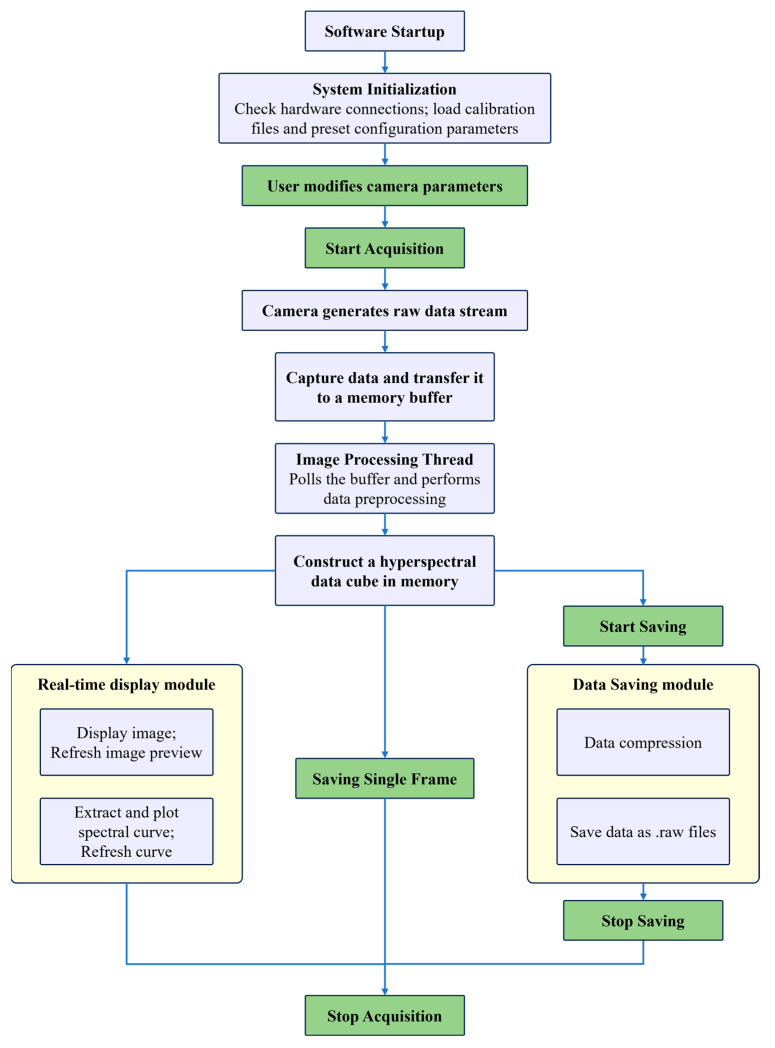
Workflow diagram of the hyperspectral data acquisition software, showing the multi-threaded operational pipeline of the custom software developed for push-broom hyperspectral imaging. The workflow integrates parameter adjustment, real-time feedback, and asynchronous data saving, supporting efficient and flexible control throughout the imaging mission.

The software achieves high-throughput data acquisition at 200 fps through a multi-threaded architecture designed to minimize blocking and maximize parallelism, supporting a data throughput as high as 500 MB/s. The detailed workflow of the software, as illustrated in Figure 9, begins with system initialization. The software automatically detects and connects to the hyperspectral camera hardware, loads predefined calibration parameters (including dark current, flat-field correction coefficients, and spectral wavelength calibration files), and configures key acquisition parameters (including integration time, frame rate, and gain). After initialization completes, the user triggers a “Start Acquisition” command. The software then sends a start signal to the camera, which enters a push-broom scanning state. The image sensor begins continuous global exposure and outputs raw digital number (DN) values. This data stream is captured in real-time via a high-speed interface (USB3.0) by a frame grabber and transferred to a designated buffer in the computer’s memory. A core processing thread within the software continuously polls this buffer. For each newly arrived frame of image data, it performs real-time dark current subtraction and flat-field correction to eliminate noise and non-uniformity. The processed hyperspectral data cube is constructed frame-by-frame in memory. Simultaneously, the software interface updates in real time: on one hand, it generates and displays a 2D spectral preview by extracting an object point, enabling users to visually assess camera overexposure in real-time. During acquisition, a separate, dedicated storage thread asynchronously writes the raw hyperspectral data stream to the hard-drive in saving order. The data is saved in the standard multi-page TIFF format. An accompanying metadata header file is automatically generated to record acquisition parameters, wavelength information, and other relevant details. When the user manually terminates data saving, the camera stops outputting data. The software finalizes the file writing process, closes the file handle, and concludes the acquisition cycle, awaiting the next start command.

**Figure 10:**
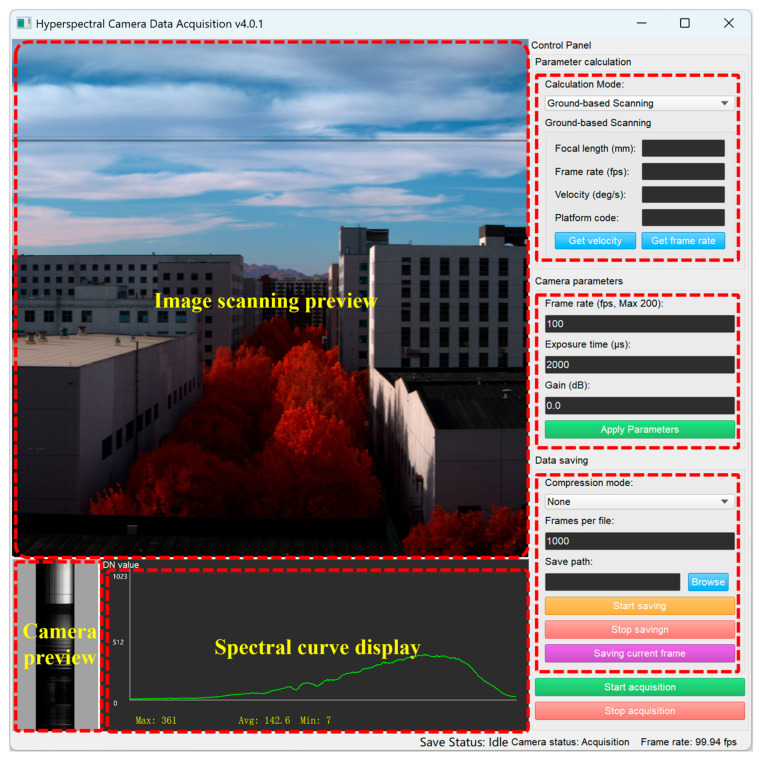
Graphical user interface of the Hyperspectral Data Acquisition Software (v4.0.1). Red boxes in dash line indicate different areas of the software interface.

The user interface of the software is shown in Figure 10. Since the hyperspectral camera operates in push-broom scanning mode, real-time preview is not intuitively straightforward. To address this, a scanning preview function was integrated into the software interface, allowing users to observe dynamically updated scene imagery during the scanning process. This enables real-time assessment of data quality and rapid adjustment of camera parameters. Additionally, a calculator tool was incorporated in the upper-right corner of the interface, supporting two operational modes: ground-based sweep scanning and aerial push-broom scanning. This feature assists users in performing on-site calculations of critical parameters such as scanning speed and frame rate.

## 3. Experimental Results

### 3.1. Assembly of Schwarzschild Imaging Spectrometer

**Figure 11:**
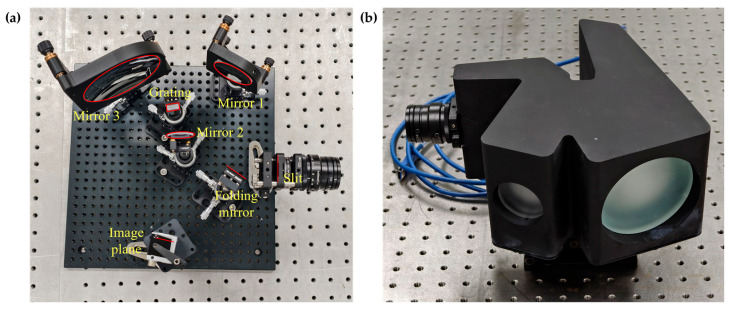
Assembling process of the Schwarzschild imaging spectrometer. (**a**) The HIS system mounted on standard threaded mounting holes on the optical platform. (**b**) The completed Schwarzschild imaging spectrometer with a 3D printed and CNC precision machined case.

After designing the whole architecture of the HSI system, the hardware was then assembled using COTS components. The implementation of the prototype strictly adheres to the Schwarzschild optical configuration, with the entire process carried out on a precision optical platform. First, to verify the feasibility of the optical design, mechanical positioning was performed according to the spatial coordinates and orientations of the components determined by the optical design according to Section 3.3. Key components, including the off-axis concave mirror (Mirror 1), convex mirror (Mirror 2), re-imaging concave mirror (Mirror 3), folding mirror, as well as the slit and image plane, were all precisely mounted onto standard threaded mounting holes on the optical platform using high-precision six-axis adjustment mounts (providing pitch, yaw, roll, and XYZ translation), as shown in Figure 11a. This ensured that the relative positions and optical axis orientations of all elements conformed to the theoretical model, laying the foundation for optical alignment.

The alignment process was critical. A reverse alignment method was adopted, using the image plane as the reference. Initially, a He-Ne laser was used as an auxiliary alignment source, temporarily placed at the designed image plane position. The laser beam was projected in reverse order: first reflecting off Mirror 2 and the Grating, then finally being collimated by Mirror 1. By observing the profile and position of the output beam, rough adjustments of the pitch and yaw angles of each mirror were made to achieve preliminary closure of the optical path. Subsequently, a real broadband light source was introduced through the slit. At this stage, fine alignment entered a critical phase: Mirror 1 was finely adjusted to ensure the collimated beam incident onto the center of the grating in parallel; the grating’s groove orientation and incident angle were then meticulously tuned so that the diffracted light were in the desired angle; finally, the pose of Mirror 2 was finely adjusted to optimize the focusing of dispersed beams of various wavelengths until a clear, sharp, and minimally distorted spectral line was formed at the image plane. The entire process required iterative optimization, with real-time monitoring at the image plane using the CMOS camera. This ensured that the imaging performance of the entire system across the full field of view and all wavebands closely approached the design specifications.

Upon completion of the opto-mechanical alignment on an optical bench, all the geometric parameters were finalized. A black housing was designed to provide structural support for the optical components while minimizing the system’s overall volume and weight, thereby enhancing its adaptability for field operation. Each optical element was fixed by adhesive bonding, wherein the optical surfaces were mounted into corresponding mounting holes, so their relative positions relied heavily on the machining accuracy of the housing. Given the structural complexity of the housing, a hybrid manufacturing approach was adopted to reduce alignment difficulty in later stages: a preliminary structure was first produced via 3D printing of high hardness nylon, followed by CNC precision machining of the mounting surfaces to ensure that positional tolerances between critical interfaces met the values specified by the tolerance analysis. The fully assembled hyperspectral camera is shown in Figure 11b.

### 3.2. Wavelength Coverage and Resolution Test

**Figure 12:**
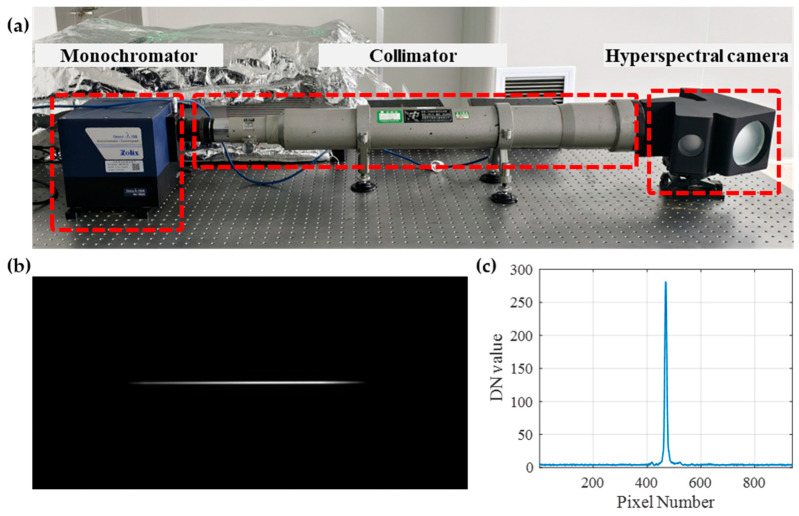
Hyperspectral calibration setup and response. (**a**) Experimental configuration for wavelength coverage and spectral resolution calibration. (**b**) Raw camera image under 702 nm monochromatic illumination, showing a distinct horizontal line response. (**c**) DN value curve of the central pixel column, with a sharp peak at pixel 470 confirming precise spectral detection.

Following the hardware integration process, wavelength coverage and spectral resolution were experimentally analyzed in the dark room. The calibration system consists of three main components: a monochromator (Omni-λ300i, Zolix Co., Ltd., Beijing, China), a collimator (F550, Changfu Technology Co., Ltd., Beijing, China), and the hyperspectral camera under test, all mounted on an optical bench to ensure mechanical and optical stability, as shown in Figure 12a.

Wavelength calibration is a critical procedure in hyperspectral imaging. Its significance lies in establishing an accurate correspondence between each pixel of the camera’s detector and a specific wavelength, which directly determines the accuracy and reliability of the acquired spectral data. In the above setup, the monochromator generates quasi-monochromatic light with narrow spectral bandwidth and continuously tunable wavelength. The output beam is collimated to form a parallel light field, simulating an object at infinity and illuminating the entrance pupil of the camera under test. The hyperspectral camera receives this monochromatic light and forms an image of the slit on its image plane. Figure 12b shows the spot distribution formed by 702 nm monochromatic light on the camera’s image plane. The spot exhibits an elongated shape with clear contours and good symmetry, which is directly related to the slit geometry and the effectiveness of astigmatism correction in the system. The high concentration of energy within the spot also reflects the excellent focusing capability of the system. This is further confirmed by the DN value curve along the central column of pixels for the 702 nm monochromatic light image, as shown in Figure 12c. A distinct peak is observed around pixel number 470, corresponding to the maximum response of the detector to the 702 nm light.

**Figure 13:**
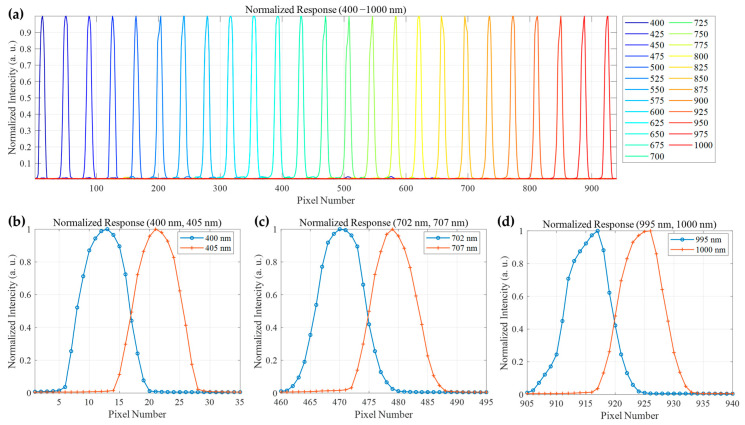
Spectral response characterization of the hyperspectral camera. (**a**) Normalized spectral response curves acquired at 25 nm intervals across the 400–1000 nm range. (**b**) Response curves for 400 nm and 405 nm illumination, showing clear separation between adjacent wavelengths, confirming a spectral resolution better than 5 nm in the short-wave region. (**c**) Corresponding curves for 702 nm and 707 nm, illustrating consistent wavelength discrimination capability in the visible-red region. (**d**) Curves for 995 nm and 1000 nm, validating performance in the near-infrared with maintained resolution.

By incrementally adjusting the output wavelength of the monochromator from 400 to 1000 nm, the digital number (DN) values along the central column of the detector were recorded and normalized for each wavelength increment. A total of 25 response curves at 25 nm intervals are plotted in Figure 13a, comprehensively covering the target wavelength range. The results demonstrate that the hyperspectral camera exhibits effective photo response throughout the entire 400–1000 nm band, with each curve showing a distinct peak corresponding to the input monochromatic light. This confirms the system’s capability for accurate spectral detection across the visible and near-infrared regions.

To quantitatively evaluate the spectral resolution, the response to adjacent monochromatic light sources with a narrow wavelength interval of only 5 nm was further analyzed. Figure 13b–d display the normalized response curves for three wavelength pairs: 400 nm and 405 nm, 702 nm and 707 nm, and 995 nm and 1000 nm, respectively. The results indicate that across different bands, i.e., short-wave (400/405 nm), mid-wave (702/707 nm), and long-wave (995/1000 nm), the response curves for all wavelength pairs are effectively separated, forming two distinct and resolvable peaks. This result demonstrates that the camera can clearly distinguish adjacent monochromatic lights with a wavelength difference of only 5 nm. According to the Rayleigh criterion, two adjacent wavelengths are considered resolved if their peak responses are clearly distinguishable and the valley between them is lower than 80% of the peak intensities. The curves for all wavelength pairs shown in the figure exceed this criterion. In conclusion, these test results proved that the developed hyperspectral camera prototype achieves a spectral coverage of 400–1000 nm with a resolution better than 5 nm across the entire operating band.

### 3.3. Acquisition of Hyperspectral Image

**Figure 14:**
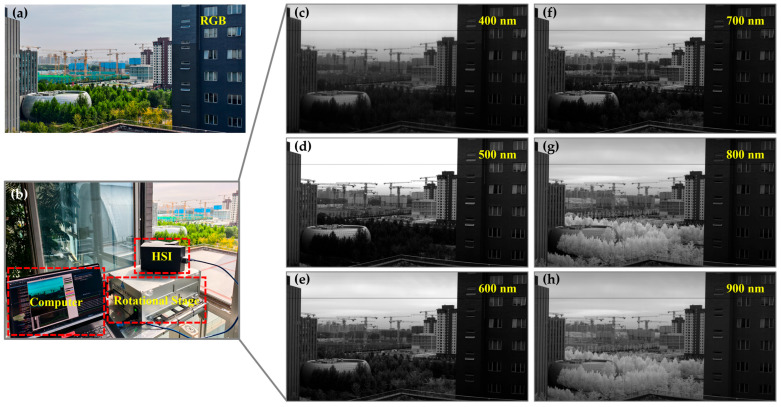
Hyperspectral imaging experimental setup and results. (**a**) True-color RGB image of an urban scene acquired with a Nikon P950 camera. (**b**) Configuration for hyperspectral camera testing, including a computer, the HSI system, and a rotational stage. (**c**–**h**) Single-band images extracted from the hyperspectral data cube at 100 nm intervals across 400–900 nm.

Following the completion of hardware integration and software development for the hyperspectral imaging system, an imaging experiment was conducted to evaluate the camera’s functionality and performance. The experiment was conducted on the 6th floor of Tower D in No. 9 Dengzhuang South Road, Haidian District, Beijing, at 10:00 a.m. under stable illumination conditions. The camera, equipped with a 25 mm focal length lens, was set to an exposure time of 5 ms and operated at a frame rate of 150 fps. It was mounted on a precision rotational stage to perform push-broom scanning of a natural scene containing various features such as trees, buildings, and the sky, while simultaneously saving data in real time. Figure 14 presents the outdoor scene imaging test results of the developed hyperspectral camera prototype. Figure 14a shows an RGB image recorded by a Nikon P950 camera (Nikon, Tokyo, Japan) with hyperspectral camera scanning, providing an intuitive visualization of the test scene: green trees, gray-white building facades, and a bright blue sky. The experimental setup, including the hyperspectral camera, the rotational stage, and the control computer, is shown in Figure 14b.

The core analysis focuses on the monochromatic band images extracted from the acquired hyperspectral data cube at central wavelengths spaced 100 nm apart (i.e., 400 nm, 500 nm, 600 nm, 700 nm, 800 nm, 900 nm) presented in Figure 14c–h. These monochromatic images clearly reveal significant brightness variations in different ground objects due to their unique spectral reflectance characteristics.

The spectral behavior of vegetation is a key focus of analysis. In the 400 nm (blue) and 500 nm (green) bands, leaves exhibit low reflectance due to strong chlorophyll absorption in the blue and red regions for photosynthesis, thus appearing dark gray in Figure 14c,d. At 600 nm (orange-red), which is near the end of the chlorophyll absorption valley (red absorption), reflectance slightly increases but remains relatively low. From 700 nm onward, a dramatic change occurs. The spongy mesophyll structure inside the leaves leads to a pronounced “near-infrared red-edge effect,” causing a sharp increase in reflectance within the 700–900 nm range. In the Figure 14f 800 nm and Figure 14h 900 nm images, the leaves appear exceptionally bright, even dazzling white, due to their very high reflectance, contrasting sharply with their appearance in other bands. This is the most distinctive spectral signature of healthy vegetation.

The spectral reflectance curve of building materials (e.g., concrete, paint) is generally flat and smooth, approximating those of a Lambertian (diffuse) reflector, exhibiting near-isotropic scattering across the measured wavelength range. This means their reflectance ability changes little across different wavelengths. Thus, in the monochromatic images from 400 nm to 900 nm, the brightness of the buildings remains relatively stable without the dramatic variations seen in vegetation. They appear as medium gray across all bands, with reflectance levels intermediate between highly reflective vegetation (in the near-infrared) and some low-reflectance dark features.

The brightness of the sky is primarily governed by atmospheric scattering mechanisms. In shorter wavelengths such as 400 nm and 500 nm, Rayleigh scattering (which is inversely proportional to the fourth power of the wavelength) is extremely significant. A large amount of blue and violet light is scattered by atmospheric molecules into the camera, making the sky appear very bright in Figure 14c,d. As the wavelength increases, Rayleigh scattering diminishes rapidly. At 600 nm and longer wavelengths, molecular scattering decreases substantially, atmospheric transmittance increases, and scattered light intensity weakens. As a result, the brightness of the sky continuously decreases in Figure 14e–h, appearing increasingly dark. This indicates that the camera successfully captured the spectral characteristics determined by atmospheric physics.

**Figure 15:**
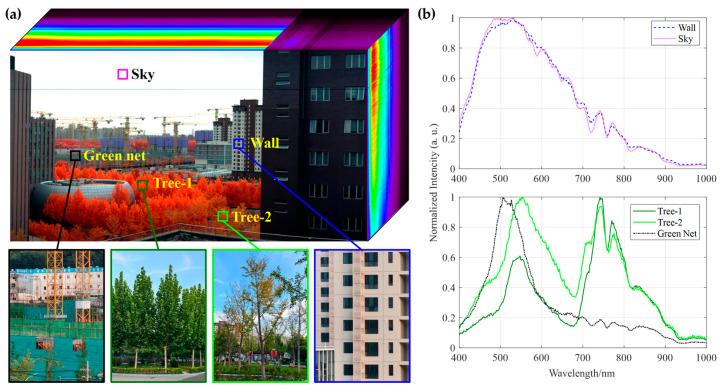
Hyperspectral data cube and spectral analysis. (**a**) Synthetic hyperspectral data cube showing a pseudo-RGB image of a construction site, including a green artificial dustproof net, building exterior wall, and two tree species: Tree-1 (*Platanus*) and Tree-2 (*Ginkgo biloba*). (**b**) Normalized DN spectral curves for five sampling points (sky, wall, dustproof net, and trees).

This experiment also successfully acquired and analyzed the three-dimensional data cube generated by the push-broom hyperspectral imaging system. The rich spectral information contained within provides critical evidence for accurately distinguishing and identifying different ground objects. As shown in Figure 15a, the data cube clearly displays typical urban features, including building facades, the sky, two types of trees, and green dust nets at a distant construction site.

Analysis of the normalized spectral curves extracted in the upper plots in Figure 15b indicates that the spectral profiles of the building facade and the sky exhibit high similarity across the entire VNIR range (400–1000 nm). Their reflectance trends with respect to wavelength are nearly identical, further confirming that the spectral property of the building façade is similar to a Lambertian (diffuse) reflector.

The spectral curves in the lower part of Figure 15b fully demonstrate the core advantage of hyperspectral technology. First, although Tree-1 (*Platanus*) and Tree-2 (*Ginkgo biloba*) both belong to vegetation and appear similar in RGB true-color imagery, their detailed spectral curves show quantifiable differences in visible-light absorption characteristics and near-infrared plateau reflectance intensity. These discrepancies may be attributed to factors such as tree species, health conditions, or canopy structure, illustrating the potential of hyperspectral data to identify intra-class spectral variability (same object, different spectra) or inter-class spectral similarity (different objects, similar spectra).

More notably, the spectral curves of the green artificial dustproof net and natural trees reveal a fundamental distinction: although their reflectance values are similar in the green band (~550 nm) due to the use of green dye mimicking chlorophyll reflectance, a dramatic difference emerges in the critical near-infrared region (>700 nm). Natural vegetation exhibits very high reflectance owing to the “near-infrared red-edge effect” caused by the internal leaf structure, whereas the artificial dust net completely lacks this bio-optical characteristic, maintaining very low reflectance. This strong contrast in the NIR region serves as a reliable spectral fingerprint for distinguishing artificial green materials from natural vegetation.

In summary, the experimental results systematically verify that the developed hyperspectral camera can effectively capture the unique spectral features of ground objects. The data cube it produces not only facilitates land cover classification but also enables fine material discrimination beyond the capability of human vision, holding significant application value in fields such as precision agriculture, environmental monitoring, and urban remote sensing.

### 3.4. Comparison with Previous Works

Having detailed the design and validation of the proposed COTS hyperspectral camera, it is crucial to benchmark these results against the state of the art. The comparative analysis that follows examines fundamental specifications such as spectral coverage, number of bands, and F-number, under relatively similar cost, highlighting the balance of performance and size realized by this work, as shown in Table 7.

Compared to previous works, this paper achieves a notable spectral coverage of 400–1000 nm VNIR range, surpassing the range of previous systems which typically covering approximately 400–800 nm. The spectral resolution of 5 nm, while slightly lower than some counterparts, results from a deliberate design choice utilizing spherical off-axis mirrors, which present challenges in high-precision alignment but offer significant advantages in cost and manufacturability. A key feature is the high number of spectral bands (930), which enhances spectral sampling density. The potential signal reduction per band inherent to such a high channel count is effectively mitigated by the high throughput of the all-reflective optical system. Furthermore, practical data volume can be optimized via pixel binning, tailoring the output to application needs without compromising utility. Critically, a performance metric not captured in the table is the integration time, directly governing frame rate. For instance, under comparable good illumination conditions (e.g., 10:30 a.m.), the system in Ref. [33] requires a 50 ms integration time, limiting its frame rate to below 20 fps. In stark contrast, the system presented here operates with a markedly shorter 5 ms integration time under similar conditions (10:00 a.m.), enabling a high frame rate of 150 fps. This substantial increase in scanning efficiency is paramount for field deployments, especially in UAV-based push-broom applications, where it directly translates to enhanced operational throughput.

## 4. Conclusions

A 400–1000 nm VNIR hyperspectral imaging camera based on a Schwarzschild configuration using COTS components and achieving a good balance between performance, size, and overall cost was developed. The all-reflective design effectively reduces optical energy loss, achieving an integration time as low as several milliseconds and a high-speed push-broom imaging at 200 fps. The system exclusively uses commercial off-the-shelf (COTS) optical and mechanical components, significantly reducing manufacturing costs and process complexity while maintaining performance. The development process systematically followed a complete workflow encompassing theoretical design, simulation verification, prototype assembly, and performance testing. The developed prototype underwent rigorous performance calibration using a monochromator–collimator standard test system. Experimental data conclusively demonstrated a spectral coverage of 400–1000 nm and a spectral resolution better than 5 nm. Imaging experiments further validated its outstanding practical performance by successfully acquiring hyperspectral data cubes of natural scenes and clearly distinguishing subtle differences in the spectral curves of various materials such as vegetation, artificial objects, and green dustproof nets. In summary, this study provides a complete hyperspectral imaging solution that is high-performance, low-cost, and engineering-ready. The developed camera shows broad application prospects in fields including precision agriculture (e.g., crop growth and pest monitoring), environmental remote sensing (e.g., water quality monitoring and ecological surveys), mineral exploration, and military target detection. Future work will focus on performing more accurate absolute and relative radiometric calibration of the system to support quantitative remote sensing inversion applications, as well as conducting long-term field experiments for specific application scenarios (e.g., precision fertilization in farmlands, water pollution monitoring) to verify its potential for operational use.

## Figures and Tables

**Table 1:** Key parameters of the Schwarzschild imaging spectrometer.

Parameters	Value
Spectral coverage	400–1000 nm
Spectral resolution	Better than 6 nm
Spectral bands	>100
Number of pixels in spatial dimension	>1200
Slit width	25 μm
Slit length	8 mm
F/#	≤4
Maximum frame rate	≥150 fps

**Table 2:** List of COTS optical components of the imaging spectrometer.

Type of Component	Parameters	Costs
Front lens	Kowa LM25HC, 1″ 25 mm 5MP C-Mount Lens (Kowa Optronics Co., Ltd., Osaka, Japan)	450 USD
Slit	Slit width 25 μm, length 12 mm, with overall size 10 × 16 × 2 mm	100 USD
Folding mirror	30 × 30 mm plane mirror, Ag coating with protective layer	25 USD
Concave mirror	R = 150 mm, D = 50 mm, Ag coating with protective layer	75 USD
Convex mirror	R = −64.55 mm, D = 30 mm, Ag coating with protective layer	150 USD
Plane blazed grating	150 L/mm, blazed wavelength 600 nm, size 15 × 25 mm	100 USD
Concave mirror	R = 150 mm, D = 100 mm, Ag coating with protective layer	100 USD
CMOS sensor module	Basler acA1920–155 μm, Sony IMX174, USB 3.0 interface, Mono	1000 USD
Total		2000 USD

**Table 3:** List of geometrical parameters of the imaging spectrometer illustrated in Figure 4. All values are calculated for the chief ray 702 nm.

Parameters	Value
*d*_1_	196.692 mm
*d*_12_	88.875 mm
*d*_23_	−30.000 mm
*d*_34_	30.000 mm
*d*_45_	−88.875 mm
*d*_5_	196.691 mm
*α*_1_	−9.869°
*α*_2_	24.000°
*α*_3_	24.000°
*α*_4_	9.869°
*α*_5_	−28.618°
*λ*	13.292°
*θ*	19.591°

**Table 4:** Initial tolerance item assignment.

System Tolerance Items	Value
Radius of Curvature Tolerance	0.02 mm
Lens/Element Spacing Tolerance	0.02 mm
Irregularity Tolerance	0.1 fringe
Element Decenter Tolerance	0.02 mm
Element Tilt Tolerance	1′

**Table 5:** Reassigned tolerance distribution results.

System Tolerance Items	Face Number	Value
Radius of Curvature Tolerance	1–2, 3–4	0.01 mm
Element Decenter Tolerance	1	0.01 mm
Element Decenter Tolerance	5	0.01 mm
Element Decenter Tolerance	1	40″
Element Decenter Tolerance	2	1.2′
Element Decenter Tolerance	4	1.2′
Element Decenter Tolerance	5	40″

**Table 6:** RMS spot radius variation and probability.

Wavelength (nm)	Nominal RMS (μm)	Possibility	ΔRMS (μm)
400	0.013795	<90%	1.38302
		<80%	0.93243
		<50%	−0.11047
702	0.011545	<90%	1.19985
		<80%	0.80361
		<50%	0.04943
1000	0.013059	<90%	2.26945
		<80%	1.57854
		50%>	0.25157

**Table 7:** Comparison of key performance of the proposed Schwarzschild imaging spectrometer with previous COTS hyperspectral systems.

Ref.	Spectral Coverage	Spectral Resolution	Mass	Number of Spectral Bands	F/#
[30]	387–801 nm	3.33 nm	1.6 kg	215	2.8
[31]	400–800 nm	3.69 nm	-	-	2.8
[33]	420–830 nm	2 nm	1.31 kg	205	3.0
This work	400–1000 nm	5 nm	1.5 kg	930	4.0

## Data Availability

The article contains the original contributions made during the study. Additional questions can be forwarded to the corresponding authors.

## Abbreviations

The following abbreviations are used in this manuscript:

CNC	computer numerical control
COTS	commercial off-the-shelf
DIY	do-it-yourself
DN	digital number
FoV	field of view
HSI	hyperspectral imaging
NA	numerical aperture
OPL	optical path length
RMS	root mean square
UAV	unmanned aerial vehicle
VNIR	visible-to-near-infrared
VPH	volume-phase holographic
